# Repeated exposure to high ICT demands at work, and development of suboptimal self-rated health: findings from a 4-year follow-up of the SLOSH study

**DOI:** 10.1007/s00420-019-01407-6

**Published:** 2019-01-25

**Authors:** Magdalena Stadin, Maria Nordin, Anders Broström, Linda L. Magnusson Hanson, Hugo Westerlund, Eleonor I. Fransson

**Affiliations:** 1 School of Health and Welfare, Jönköping University, P.O. Box 1026, 551 11 Jönköping, Sweden; 2 Stress Research Institute, Stockholm University, Stockholm, Sweden; 3 Department of Psychology, Umeå University, Umeå, Sweden; 4 Department of Clinical Neurophysiology, Linköping University Hospital, Linköping, Sweden

**Keywords:** ICT demands at work, Occupational health, Work-related stress, Self-rated health, Gender differences, Socioeconomic position

## Abstract

**Purpose:**

The knowledge about the association between Information and Communication Technology (ICT) demands at work and self-rated health (SRH) is insufficient. The aim of this study was to examine the association between repeated exposure to high ICT demands at work, and risk of suboptimal SRH, and to determine modifications by sex or socioeconomic position (SEP).

**Methods:**

A prospective design was used, including repeated measurement of ICT demands at work, measured 2 years apart. SRH was measured at baseline and at follow-up after 4 years. The data were derived from the Swedish Longitudinal Occupational Survey of Health (SLOSH), including 4468 gainfully employees (1941 men, 2527 women) with good SRH at baseline.

**Results:**

In the total study sample, repeated exposure to high ICT demands at work was associated with suboptimal SRH at follow-up (OR 1.34 [CI 1.06–1.70]), adjusted for age, sex, SEP, health behaviours, BMI, job strain and social support. An interaction between ICT demands and sex was observed (*p* = 0.010). The risk was only present in men (OR 1.53 [CI 1.09–2.16]), and not in women (OR 1.17 [CI 0.85–1.62]). The risk of suboptimal SRH after consistently high ICT demands at work was most elevated in participants with high SEP (OR 1.68 [CI 1.02–2.79]), adjusted for age, sex, health behaviours, BMI and job strain. However, no significant interaction between ICT demands and SEP regarding SRH was observed.

**Conclusion:**

Repeated exposure to high ICT demands at work was associated with suboptimal SRH at follow-up, and the association was modified by sex.

## Introduction

Modern working life is characterised by digitalisation, including use of information and communication technology (ICT) (Swedish Work Environment Authority 2015a). The concept of ICT is a modification of information technology (IT), and refers to technologies that provide access to information and communication by communicative technical devices, such as the Internet, tablets, and smartphones (Christensen 2017). In Sweden, 93% of the working population have access to the Internet through their work place, and 50% are using tablets or smartphones in their work daily (Swedish Work Environment Authority 2015a; The Internet Foundation in Sweden 2018). ICT facilitates many job tasks, provides more flexible work and may contribute to increased work efficiency (Barber and Jenkins 2014; Chesley 2014; Swedish Work Environment Authority 2015a). On the other hand, aspects such as a high amount of emails and telephone calls, expectations of high availability via ICT devices, and problems with ICT devices that do not work properly, are all potential stressors in the modern industrialised work environment (Barber and Santuzzi 2015; Day et al. 2012; Stadin et al. 2016; Stenfors et al. 2013).

ICT-related stress has been associated with poor health-related outcomes such as suboptimal (i.e., below the optimal standard) general self-rated health (SRH), cognitive complaints involving problems with concentration, memory, decision-making and ability to think clearly, sleep disturbances, burnout and sickness absenteeism (Barber and Jenkins 2014; Barber and Santuzzi 2015; Hennington et al. 2011; Stadin et al. 2016; Stenfors et al. 2013). However, the knowledge about ICT-related stress and its association with different health-related outcomes over time is still very limited, and additional studies have been called for (Swedish Work Environment Authority 2015a).

The association between work-related stress (including ICT demands) and health-related outcomes can be understood by the job-demands-resources (JD-R) model (Bakker and Demerouti 2007). This model claims that the association between job demands and health is modified by different types of resources (e.g., individual and organisational). Two well-known models used in research regarding work-related stress and health are the demand-control (job strain) model (Karasek and Theorell 1992) and the effort-reward imbalance model (Siegrist et al. 1997). Both these two models fit under the more general JD-R model.

The association between work-related stress in general and health is modified by sex, and women have for instance a higher prevalence of work-related stress operationalised as job strain, and are absent due to stress-related disorders to a greater extent than men (Swedish Social Insurance Agency 2016; Swedish Work Environment Authority 2016). This can partly be explained by the sex segregated labour market, including a structural uneven distribution of men and women in different occupations and sectors, and job positions within the work sectors (Ellingsæter 2014; Swedish Work Environment Authority 2016). However, less is known about the possible influence by sex on the association between ICT-related stress at work and health-related outcomes.

Social gradients in general indicators of work-related stress and its association with health-related outcomes are a well-investigated phenomenon, and people in lower socioeconomic positions (SEP) have in general a higher frequency of work-related stress that predicts poor health-related outcomes (Hoven and Siegrist 2013; Swedish Work Environment Authority 2015b; Toivanen 2011). This pattern can theoretically be explained by the ‘status syndrome’, that implies that the social standing predicts the health and longevity, mostly due to a lower degree of control over the working situation in the lower SEP groups (Marmot 2004). However, when it comes to ICT-related stress at work and SEP, the knowledge is limited, but cross-sectional analyses have shown a higher frequency of high ICT demands at work, among people with intermediate and high SEP (Stadin et al. 2016), which may indicate that the association between ICT-related stress and SEP might differ from the association between general indicators of work-related stress and SEP.

There is a lack of studies that are examining the prospective association between ICT-related stress at work and health-related outcomes. The knowledge about the possible influence by sex and SEP on the association between ICT-related stress at work and health-related outcomes is also limited. Consequently, the aim of this study was to examine the prospective association between ICT-related stress operationalised as high ICT demands at work, and its association with suboptimal SRH in a working population in Sweden, and to examine if such an association differs by sex or by SEP. In addition, repeated measurement of high ICT demands at work will be used, since previous findings imply that repeated exposure to stressors may have a stronger association with poor health-related outcomes, than single exposure only (Somville et al. 2016; Yuen et al. 2012).

## Materials and methods

### Procedure and participation

Data from the Swedish Longitudinal Occupational Survey of Health (SLOSH) were used. The overall aim of SLOSH is to examine associations between work participation, work environment, social situation and health/wellbeing (Magnusson Hanson et al. 2015; Magnusson Hanson et al. 2008; Statistics Sweden 2014). SLOSH is an ongoing cohort study using biennial questionnaires, which first started in 2006. The participants are previous respondents to the Swedish Work Environment Survey, which aims to comprise a representative sample of the working population in Sweden. The response rate at baseline 2006 was 65.0% and generated an initial sample of 9214 respondents. In the data collection of 2008, 9639 new responders were recruited, representing a response rate of 61.1%. Further details of the data collections and participation in SLOSH have been published elsewhere (Magnusson Hanson et al. 2015; Magnusson Hanson et al. 2008; Statistics Sweden 2014). To be acknowledged, in the present study, only respondents that responded to the questionnaire at three data collections (baseline either 2006 or 2008), 2 years apart, and remained gainfully employed during the whole period, were included in the analytical study sample.

### Analytical study sample

**Fig. 1:**
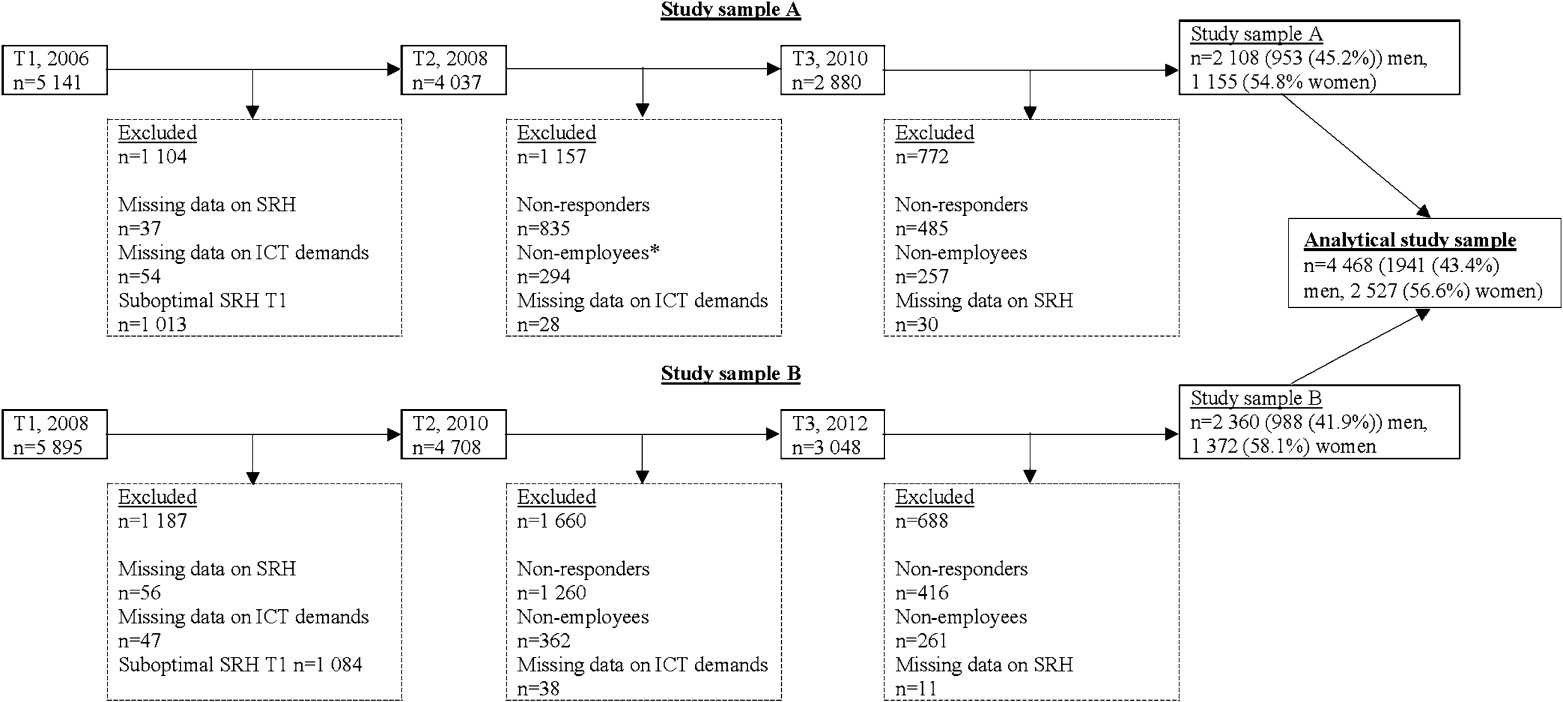
Analytical study sample. *Note* “Non-employee” refers to people who are unemployed, on long-term sick leave, homeworkers, retired etc

In the present study, SLOSH data collected in 2006, 2008, 2010 and 2012 were used. Based on those data collections, two study samples were created, study samples A (baseline at 2006) and B (baseline at 2008) (Fig. 1). To increase the size of the analytical sample in the present study, we merged samples A and B into one. Out of those, we excluded respondents who had not responded to all three measurements, were non-employees at any of the analysed measurements (e.g., retired), reported suboptimal SRH at T1 or had missing data on ICT demands at work at T1 or T2, or missing data on SRH at T1 or T3. This left 4468 gainfully employed persons (1941 [43.4%] men and 2527 [56.6%] women) as the analytical study sample (Fig. 1). The data collection of SLOSH 2014 was not included because the ICT demands at work scale was excluded in SLOSH 2012, and the latter could consequently only be used as a follow-up of SRH in the present study.

### ICT demands at work

ICT demands at work were measured by a scale specifically developed for SLOSH (Stadin et al. 2016; Stenfors et al. 2013), based on previous work by Johansson-Hidén et al. (2003). The scale is introduced as follows: ‘New technology and more flexible working conditions have changed working life for many people. Technology can be very helpful but is also conducive to new types of stress. Estimate to what extent you are stressed by…’. Then follow five items in the 2006 data collection; ‘…too many calls and emails’, ‘…claims to be available on work-related issues both during work hours and leisure time’, ‘…claims to give immediate answers to emails and telephone calls that require a lot of work’, ‘…constantly being interrupted by the telephone and email’, and ‘…computers and other equipment that fail to work properly’. In the 2008 data collection, the item ‘…claims to be available on work-related issues both during work hours and leisure time’ was split into two items, separately focusing on either work hours or leisure time. Cronbach’s alpha of the ICT demands at work scale was measured at T1, and the analysis was conducted separately in group A (0.89) and group B (0.87), respectively. The response options were rated on a Likert scale from 1 (I do not have access to this at work) to 5 (very much). ICT demands at work was calculated as the mean score of the ICT demand items. The median score of ICT demands at work (3.00 at both T1 and T2) was used as the cut-off value for high and low ICT demands at work (high ICT demands was defined as strictly above the median) across all measurements. ‘Non-exposure T1, T2’ (low ICT demands at both T1 and T2), was compared with ‘exposure T1, non-exposure T2’ (high ICT demands at T1, and low ICT demands at T2), ‘non-exposure T1, exposure T2’ (low ICT demands at T1, and high ICT demands T2) and ‘exposure T1 and T2’ (high ICT demands at T1 and T2).

### Self-rated health

Self-rated health refers to the general subjective health status, and was measured by the one-item question: ‘How would you rate your general state of health?’, which originally was introduced in the SF-36 scale (Sullivan et al. 1995). The response options were rated on a Likert scale from 1 (very bad) to 5 (very good). In the analyses, suboptimal SRH (defined as responding ‘very bad’, ‘rather bad’, or ‘neither good nor bad’) was contrasted to good SRH (defined as responding ‘quite good’ or ‘very good’ to the question).

### Covariates

Age, sex, SEP, health behaviours, Body Mass Index (BMI), job strain, and social support were treated as potential confounding factors. These factors have been included based on previous associations with different indicators of work-related stress and SRH (Moor et al. 2016; Rydstedt et al. 2012; Stadin et al. 2016; Stenholm et al. 2017; Toivanen 2011). SEP was calculated in three categories; ‘low SEP’ (unskilled, semiskilled and skilled workers), ‘intermediate SEP’ (assistant and intermediate non-manual workers) and ‘high SEP’ (employed and self-employed professionals, higher civil servants and executives), classified in line with Statistics Sweden’s manual of the socioeconomic classification (Statistics Sweden 1982). Age was calculated in four categories, ‘20–39 years’, ‘40–49 years’, ‘50–59 years’ and ‘60–68 years’. Smoking was calculated in two categories; ‘smoking’ (daily and occasionally) and ‘non-smoking’. Physical activity during leisure time was measured by the question ‘How much do you practise physical exercise?’ The answers were rated on a four-grade ordinal scale from ‘never exercise’ to ‘exercise regularly’, which was dichotomised into the categories ‘low and occasional physical activity’ and ‘regular physical activity’. BMI was calculated by self-reported weight in kilograms/height in squared meters and classified into four categories; ‘underweight’ (< 18.50), ‘normal weight’ (18.50–24.99), ‘overweight’ (25.00–29.99) and ‘obesity’ (≥ 30.00) (World Health Organization 2015).

Job strain was calculated by the demand-control questionnaire (DCQ), which covers the dimensions ‘demands’, based on five items, e.g. ‘Does your work demand too much effort?’, and ‘control’, based on six items, e.g. ‘Do you have a choice in deciding what you do at work?’ (Karasek and Theorell 1992). The population medians of the demands (2.60) and control dimensions (3.17) at T1 were used as cut-off values for high or low scores of the dimensions. ‘Job strain’ was calculated in the categories ‘job strain’ (combination of high demands (strictly above the median), and low control (strictly below the median)) and ‘no strain’ (all other combinations of the demand and control dimensions).

Social support was measured by the social support dimension in the demand-control-support questionnaire (DCSQ), that is based on six items, e.g. ‘I get on well with my colleagues’ (Chungkham et al. 2013). The population median at T1 (1.83) was used as cut-off value for high or low score of social support. Social support was calculated in the categories ‘low social support’ (strictly below the median), and ‘high social support’ (equal to or above the median).

Sex and SEP were also treated as potential effect modifiers. This was due to previous findings of sex differences in diverse work characteristics and health-related outcomes (Swedish Work Environment Authority 2016), and findings indicating that SEP modifies the association between work-related stress and health-related outcomes (Hoven and Siegrist 2013; Toivanen 2011). Information about all potential confounding or modifying factors was included in the SLOSH questionnaire and measured at T1.

### Statistical analyses

Chi-square tests were conducted for bivariate analyses to test potential differences with regard to study sample A or B, sex and SEP in the prevalence of high ICT demands at work, and other characteristics. Logistic regression analyses, calculating odds ratios (OR) with 95% confidence intervals (CI) were used to examine the association between repeated exposure to ICT demands at work and development of suboptimal SRH at follow-up. Repeated exposure to ICT demands at work in relation to suboptimal health was analysed in a crude analysis, and in four different multivariable adjusted regression models: Model 1 was adjusted for age, sex and SEP, Model 2 was adjusted for age, sex, SEP, health behaviours and BMI, Model 3 was adjusted for age, sex, SEP, health behaviours, BMI and job strain, and Model 4 was adjusted for age, sex, SEP, health behaviours, BMI, job strain and social support. All analyses were carried out in the total study sample, and stratified by sex and SEP, separately. Tests for statistical interaction between ICT demands at work and sex, and ICT demands at work and SEP, were also conducted, by including a statistical interaction term between ICT demands and sex or SEP in the respective logistic regression model. As sensitivity analyses, the main results were calculated separately in study samples A and B, and additionally calculated on a modified study sample, that excluded participants stating that they did not have access to ICT at work. The alpha was set to < 0.05. IBM SPSS Statistics 21 was used to calculate the results.

## Results

### Characteristics in the total study sample

The total study population comprised 4468 participants (Fig. 1), 1941 (43.4%) men and 2527 (56.6%) women. Out of the 4273 partcipants with adequate information about SEP, 1259 (29.4%) were categorised into low SEP 2024 (47.4%) into intermediate SEP and 990 (23.2%) into high SEP (Table 1). Most of the participants were between 40 and 59 years, and the mean age was 47.3 years. Concerning exposure to high ICT demands at work, 43.9% of the participants were unexposed to high ICT demands at work at both T1 and T2; 26.1% were exposed to high ICT demands at work demands at either T1 or T2; and 30.0% were exposed to high ICT demands at work at both T1 and T2. Job strain was prevalent in 16.1% and 46.2% reported low social support (Table 1).

**Table 1:** Characteristics in the total study sample, and in study sample A (baseline 2006) and B (baseline 2008)

Characteristics	Total	Study sample A	Study sample B	*p* value*
	*n* = 4468	*n* = 2108	*n* = 2360	
Age, *n* (%)				
20–39 years	970 (21.2)	480 (22.8)	473 (20.0)	ns
40–49 years	1494 (32.6)	669 (31.7)	799 (33.9)	
50–59 years	1753 (38.2)	805 (38.2)	899 (38.1)	
60–68 years	368 (8.0)	154 (7.3)	189 (8.0)	
Sex, *n* (%)				
Men	1941 (43.4)	953 (45.2)	988 (41.9)	0.024
Women	2527 (56.6)	1155 (54.8)	1372 (58.1)	
SEP, *n* (%)				
Total	4273 (100.0)	2022 (47.3)	2251 (52.7)	
Low	1259 (29.4)	609 (30.1)	650 (28.9)	ns
Intermediate	2024 (47.4)	955 (47.2)	1069 (47.5)	
High	990 (23.2)	458 (22.7)	532 (23.6)	
ICT demands at work, *n* (%)				
Non-exposure T1, T2	1959 (43.9)	943 (44.7)	1016 (43.1)	0.004
Exposure T1, non-exposure T2	584 (13.1)	238 (11.3)	346 (14.7)	
Non-exposure T1, Exposure T2	583 (13.0)	296 (14.0)	287 (12.2)	
Exposure T1 & T2	1342 (30.0)	631 (29.9)	711 (30.1)	
Job strain, *n* (%)				
No strain	3733 (83.9)	1765 (84.2)	1968 (83.6)	ns
Job strain	718 (16.1)	332 (15.8)	386 (16.4)	
Social support, *n* (%)				
Low social support	2090 (46.2)	943 (45.2)	1103 (47.2)	ns
High social support	2438 (53.8)	1142 (54.8)	1234 (52.8)	
Smoking, *n* (%)				
Non-smoking	3880 (87.1)	1825 (86.8)	2055 (87.3)	ns
Smoking	577 (12.9)	277 (13.2)	300 (12.7)	
Physical activity, *n* (%)				
Low/occasional	2145 (48.3)	1037 (49.3)	1108 (47.3)	ns
Regular	2300 (51.7)	1066 (50.7)	1234 (52.7)	
BMI, *n* (%)				
<18.50	34 (0.8)	14 (0.7)	20 (0.9)	ns
18.50-24.99	2295 (52.1)	1103 (53.1)	1192 (51.2)	
25.00-29.99	1661 (37.7)	779 (37.5)	882 (37.9)	
≥30.00	413 (9.4)	180 (8.7)	233 (10.0)	

BMI < 18.50 = underweight; BMI 18.50–24.99 = normal weight; BMI 25.00–29.99 = verweight; BMI ≥ 30.00  = obesity

*ns* non-significant

*Chi-square test for comparison of proportions

### Characteristics in study sample A and B

Study sample A comprised 2108 participants, and study sample B comprised 2360 participants (Table 1). A higher proportion of women than men participated in both study sample A and B, but the proportion of women (58.1%) was greater in study sample B compared with study sample A (54.8%). Minor differences between study sample A and B were found considering exposure to ICT demands at work. Among those participants that were exposed to high ICT demands at work at only one time-point, it was more prevalent to be exposed at T1 in study sample B (14.7%) than in study sample A (11.3%). Correspondingly, it was more prevalent to only be exposed to high ICT demands at work at T2 in study sample A (14.0%) than in study sample B (12.2%). No difference between study sample A and B was observed considering age, SEP, job strain, social support, health behaviours and BMI (Table 1).

### Characteristics of men and women

Men reported slightly lower exposure to high ICT demands at work in all categories (exposure at T1, exposure at T2, and exposure at T1 and T2) as compared to women (Table 2). Job strain was observed to be more prevalent in women (18.1%), than in men (13.6%). No sex difference was observed regarding social support. Sex differences were observed in health behaviours and BMI (Table 2).

**Table 2:** Characteristics in men and women, and in participants with low, intermediate and high SEP

Characteristics	Men	Women	*p* value*	Low SEP	Intermediate SEP	High SEP	*p* value*
	*n* = 1941	*n* = 2527		*n* = 1259	*n* = 2024	*n* = 990	
Age, *n* (%)							
20–39 years	452 (22.8)	518 (19.9)	< 0.001	265 (20.3)	433 (21.0)	241 (24.0)	0.001
40–49 years	607 (30.6)	887 (34.1)		412 (31.6)	695 (33.7)	321 (31.9)	
50–59 years	739 (37.2)	1014 (39.0)		532 (40.8)	801 (38.8)	343 (34.1)	
60–68 years	187 (9.4)	181 (7.0)		95 (7.3)	135 (6.5)	100 (10.0)	
Sex, *n* (%)							
Men	–	–	–	649 (51.5)	675 (33.3)	483 (48.8)	< 0.001
Women	–	–		610 (48.5)	1349 (66.7)	507 (51.2)	
SEP, *n* (%)							
Total	1807 (42.3)	2466 (57.7)	< 0.001				
Low	649 (35.9)	610 (24.7)	< 0.001	–	–	–	–
Intermediate	675 (37.4)	1349 (54.7)		–	–	–	
High	483 (26.7)	507 (20.6)		–	–	–	
ICT demands at work, *n* (%)							
Non-exposure T1, T2	890 (45.9)	1069 (42.3)	0.037	835 (66.3)	742 (36.7)	310 (31.3)	< 0.001
Exposure T1, non-exposure T2	246 (12.7)	338 (13.4)		147 (11.7)	288 (14.2)	127 (12.8)	
Non-exposure T1, Exposure T2	227 (11.7)	356 (14.1)		119 (9.5)	275 (13.6)	157 (15.9)	
Exposure T1 & T2	578 (29.8)	764 (30.2)		158 (12.5)	719 (35.5)	396 (40.0)	
Job strain, *n* (%)							
No strain	1670 (86.4)	2063 (81.9)	< 0.001	972 (77.5)	1688 (83.7)	898 (91.0)	< 0.001
Job strain	263 (13.6)	455 (18.1)		282 (22.5)	329 (16.3)	89 (9.0)	
Social support, *n* (%)							
Low social support	931 (47.6)	1159 (45.0)	ns	634 (49.1)	951 (46.3)	440 (44.4)	ns
High social support	1023 (52.4)	1415 (55.0)		657 (50.9)	1101 (53.7)	551 (55.6)	
Smoking, *n* (%)							
Non-smoking	1715 (88.6)	2165 (85.9)	0.008	1028 (81.9)	1783 (88.3)	906 (91.7)	< 0.001
Smoking	221 (11.4)	356 (14.1)		227 (18.1)	237 (11.7)	82 (8.3)	
Physical activity, *n* (%)							
Low/occasional	1088 (56.3)	1057 (42.1)	< 0.001	703 (56.1)	907 (45.1)	429 (43.6)	< 0.001
Regular	844 (43.7)	1456 (57.9)		550 (43.9)	1106 (54.9)	555 (56.4)	
BMI, *n* (%)							
<18.50	4 (0.2)	30 (1.2)	< 0.001	6 (0.5)	18 (0.9)	10 (1.0)	< 0.001
18.50–24.99	776 (40.3)	1519 (61.3)		545 (44.2)	1094 (54.8)	561 (57.1)	
25.00–29.99	955 (49.6)	706 (28.5)		532 (43.1)	704 (35.3)	346 (35.2)	
≥30.00	190 (9.9)	223 (9.0)		150 (12.2)	179 (9.0)	65 (6.6)	

BMI < 18.50 = underweight; BMI 18.50–24.99 = normal weight; BMI 25.00–29.99 = overweight; BMI ≥ 30.00 = obesity

*ns* non-significant

*Chi-square test for comparison of proportions

### Characteristics of participants with low, intermediate and high SEP

SEP differences were found in both ICT demands at work and job strain (Table 1). Repeated exposure to high ICT demands at work (measured at T1 and T2) was more common among participants with high SEP (40.0%), followed by participants with intermediate SEP (35.5%) and low SEP (12.5%). Job strain was observed to be more prevalent in participants with low SEP (22.5%), followed by participants with intermediate SEP (16.3%) and high SEP (9.0%). No SEP difference was observed in social support. SEP differences were found in health behaviours and in BMI (Table 1).

### ICT demands at work and risk of developing suboptimal self-rated health, total study sample

Repeated exposure to high ICT demands at work was associated with increased risk of suboptimal SRH in the crude analysis (OR 1.36 [CI 1.11–1.67]), and also after adjustments for age, sex, SEP, health behaviours, BMI, job strain and social support (OR 1.34 [CI 1.06–1.70]) (Table 3). No associations were observed between exposure at one point in time (either T1 or T2) and suboptimal health at follow-up.

**Table 3:** Association between repeated exposure to high ICT demands at work at T1 and/or T2, and risk of developing suboptimal SRH at T3, in the total study sample and in men and women

	Crude		Adjusted for age, sex^a^ and SEP		Adjusted for age, sex^a^, SEP, health behaviours and BMI		Adjusted for age, sex^a^, SEP, health behaviours, BMI and job strain		Adjusted for age, sex^a^, SEP, health behaviours BMI, job strain and social support	
	OR	CI 95%	OR	CI 95%	OR	CI 95%	OR	CI 95%	OR	CI 95%
Total study sample										
*Exposure to ICT demands at work*	*n* = 4468		*n* = 4273		*n* = 4184		*n* = 4170		*n* = 4137	
Non-exposure T1, T2 (*n* = 1959)	1	(Ref)	1	(Ref)	1	(Ref)	1	(Ref)	1	(Ref)
Exposure T1, non-exposure T2 (*n* = 584)	0.99	0.74–1.32	1.03	0.76–1.39	1.03	0.76–1.40	1.02	0.75–1.39	1.01	0.74–1.37
Non-exposureT1, exposure T2 (*n* = 583)	0.86	0.63–1.16	0.94	0.68–1.28	0.93	0.67–1.28	0.93	0.68–1.29	0.93	0.67–1.29
Exposure T1 & T2 (*n* = 1342)	1.36	1.11–1.67	1.45	1.16–1.81	1.41	1.12–1.77	1.38	1.09–1.74	1.34	1.06–1.70
Interaction ICT demands*sex, *p* value	0.010		0.010		0.056		0.041		0.043	
Men	*n* = 1941		*n* = 1807		*n* = 1781		*n* = 1774		*n* = 1760	
Non-exposure T1, T2 (*n* = 890)	1	(Ref)	1	(Ref)	1	(Ref)	1	(Ref)	1	(Ref)
Exposure T1, non-exposure T2 (*n* = 246)	1.09	0.72–1.67	1.10	0.70–1.71	1.05	0.67–1.65	1.06	0.67–1.67	1.04	0.66–1.64
Non-exposureT1, exposure T2 (*n* = 227)	1.20	0.79–1.84	1.33	0.85–2.09	1.32	0.84–2.08	1.36	0.86–2.15	1.37	0.86–2.16
Exposure T1 & T2 (*n* = 578)	1.49	1.11–2.01	1.66	1.20–2.31	1.57	1.12–2.20	1.59	1.14–2.24	1.53	1.09–2.16
Women	*n* = 2527		*n* = 2466		*n* = 2403		*n* = 2396		*n* = 2377	
Non-exposure T1, T2 (*n* = 1069)	1	(Ref)	1	(Ref)	1	(Ref)	1	(Ref)	1	(Ref)
Exposure T1, non-exposure T2 (*n* = 338)	0.91	0.61–1.36	0.96	0.64–1.45	1.00	0.66–1.51	0.97	0.64–1.46	0.97	0.64–1.47
Non-exposureT1, exposure T2 (*n* = 356)	0.64	0.41–1.00	0.68	0.43–1.06	0.65	0.41–1.05	0.65	0.40–1.03	0.64	0.40–1.03
Exposure T1 & T2 (*n* = 764)	1.27	0.96–1.68	1.28	0.94–1.74	1.25	0.91–1.72	1.19	0.86–1.64	1.17	0.85–1.62

^a^Sex was only adjusted for in the analyses of the total study population

### ICT demands at work and risk of developing suboptimal self-rated health in men and women

The sex stratified analyses showed that repeated exposure to high ICT demands at work was associated with increased risk of suboptimal SRH among men ((OR 1.49 [CI 1.11–2.01]), crude analysis) (Table 3). This association remained after adjustment for age, SEP, health behaviours, BMI, job strain and social support (OR 1.53 [CI 1.09–2.16]). The OR was lower and not statistically significant among women (OR 1.17 [CI 0.85–1.62]), adjusted for age, SEP, health behaviours, BMI, job strain and social support. A test for statistical interaction between ICT demands at work and sex in the total study population, was statistically significant in all models, except the model adjusted for age, SEP, health behaviours and BMI, where the *p* value for interaction was 0.056 (Table 3).

### ICT demands at work and risk of developing suboptimal self-rated health in different SEP strata

The SEP-stratified crude analysis showed that repeated exposure to high ICT demands at work was associated with increased risk of developing suboptimal SRH among participants with high SEP (OR 1.76 [CI 1.10–2.84]), followed by participants with low SEP (OR 1.61 [CI 1.03–2.52]) (Table 4). When the analyses were additionally adjusted for age, sex, health behaviours, BMI, job strain and social support, the OR among participants with low SEP was slightly increased (OR 1.67 [CI 1.04–2.66]) but attenuated and was not statistically significant among participants with high SEP (OR 1.56 [CI 0.94–2.60]). The risk was lower and not significant among participants with intermediate SEP either in the crude analysis (OR 1.24 [CI 0.91–1.69]) or in the analysis adjusted for age, sex, health behaviours, BMI, job strain and social support (OR 1.07 [CI 0.77–1.49]). A test for statistical interaction between ICT demands at work and SEP on the total study population, was not statistically significant in any of the regression models (Table 4).

**Table 4:** Association between repeated exposure to high ICT demands at work at T1 and/or T2, and risk of developing suboptimal SRH at T3, in participants with low, intermediate and high SEP

	Crude		Adjusted for age and sex		Adjusted for age, sex, health behaviours and BMI		Adjusted for age, sex, health behaviours, BMI and job strain		Adjusted for age, sex, health behaviours, BMI, job strain and social support	
	OR	CI 95%	OR	CI 95%	OR	CI 95%	OR	CI 95%	OR	CI 95%
Low SEP										
*Exposure to ICT demands at work*	*n* = 1259		*n* = 1259		*n* = 1227		*n* = 1222		*n* = 1212	
Non-exposure T1, T2 (*n* = 835)	1	(Ref)	1	(Ref)	1	(Ref)	1	(Ref)	1	(Ref)
Exposure T1, non-exposure T2 (*n* = 147)	1.28	0.78–2.08	1.29	0.79–2.12	1.37	0.83–2.26	1.37	0.83–2.27	1.37	0.83–2.27
Non-exposure T1, exposure T2 (*n* = 119)	1.07	0.61–1.88	1.09	0.62–1.93	1.14	0.64–2.03	1.16	0.65–2.07	1.21	0.68–2.17
Exposure T1 & T2 (*n* = 158)	1.61	1.03–2.52	1.63	1.04–2.56	1.65	1.04–2.62	1.66	1.04–2.64	1.67	1.04–2.66
Interaction ICT demands*SEP, *p* value (Total study sample)	0.110		0.176		0.257		0.293		0.271	
Intermediate SEP	*n* = 2024		*n* = 2024		*n* = 1982		*n* = 1976		*n* = 1966	
Non-exposure T1, T2 (*n* = 742)	1	(Ref)	1	(Ref)	1	(Ref)	1	(Ref)	1	(Ref)
Exposure T1, non-exposure T2 (*n* = 288)	0.95	0.61–1.46	0.95	0.61–1.47	0.93	0.60–1.45	0.87	0.55–1.36	0.86	0.55–1.35
Non-exposure T1, exposure T2 (*n* = 275)	0.72	0.44–1.16	0.72	0.44–1.16	0.66	0.40–1.10	0.65	0.39–1.08	0.64	0.39–1.07
Exposure T1 & T2 (*n* = 719)	1.24	0.91–1.69	1.22	0.90–1.67	1.15	0.84–1.59	1.09	0.78–1.51	1.07	0.77–1.49
High SEP	*n* = 990		*n* = 990		*n* = 975		*n* = 972		*n* = 959	
Non-exposure T1, T2 (*n* = 310)	1	(Ref)	1	(Ref)	1	(Ref)	1	(Ref)	1	(Ref)
Exposure T1, non-exposure T2 (*n* = 127)	0.77	0.35–1.68	0.76	0.35–1.66	0.70	0.32–1.57	0.73	0.33–1.63	0.70	0.31–1.57
Non-exposureT1, exposure T2 (*n* = 157)	1.22	0.65–2.31	1.23	0.65–2.33	1.23	0.64–2.37	1.29	0.66–2.50	1.27	0.65–2.46
Exposure T1 & T2 (*n* = 396)	1.76	1.10–2.84	1.75	1.08–2.83	1.64	0.99–2.69	1.68	1.02–2.79	1.56	0.94–2.60

SEP was only adjusted for in the analyses of the total study population

### Sensitivity analyses

In analyses separating study samples A and B, both study samples showed ORs that were higher in the groups with repeated exposure to high ICT demands at work, than in the groups with single exposure to high ICT demands at work. However, the risk of developing suboptimal SRH after consistently high ICT demands at work was somewhat more pronounced in study sample B (OR 1.57 [CI 1.16–2.14]), than in study sample A (OR 1.33 [CI 0.96–1.84]), adjusted for age, sex, and SEP. In analyses excluding participants that stated that they did not have access to ICT at work, no obvious difference in the association between repeated exposure to high ICT demands at work and risk of suboptimal SRH was observed among these (OR 1.35 [CI 1.07–1.72]), in comparison with the original total study sample (OR 1.34 [CI 1.06–1.70]), adjusted for age, sex, SEP, health behaviours, BMI, job strain and social support.

## Discussion

In the present study, the prospective association between high ICT demands at work, measured at two points in time, and the risk of developing suboptimal SRH at follow-up, 4 years after baseline was examined. Whether this association was modified by sex or by SEP was also examined. The results showed that repeated exposure to high ICT demands at work was associated with increased risk of developing suboptimal SRH at follow-up. This risk was only present in men, and an interaction effect between ICT demands at work and sex was observed. Concerning SEP, consistently high ICT demands at work were most prevalent among participants with high SEP, even though no statistical significant interaction between ICT demands at work and SEP with regard to SRH was observed.

The results in the present study also strengthens the hypothesis that high ICT demands at work increase the risk of suboptimal SRH, which previously has been observed in cross-sectional analyses (Stadin et al. 2016), and are in line with the JD-R model (Bakker and Demeroutu 2007). However, in the present study, this risk was only present at repeated exposure to high ICT demands at work and not at single exposure to high ICT demands at work. This result, along with the previous cross-sectional findings of associations between high ICT demands at work and suboptimal SRH (Stadin et al. 2016), may indicate that the impact ICT demands at work have on SRH either is rather short-term, or require consistently exposure to high ICT demands at work. Hypothetically, the potential lack of recovery during and after work in the group with repeated exposure to high ICT demands at work may have contributed to the increased risk of suboptimal SRH in that group (Barber and Santuzzi 2015; Geurts and Sonnentag 2006). This result is also in line with previous findings that repeated exposure to stressors may have a stronger effect on health-related outcomes than exposure on a single occasion (Somville et al. 2016; Yuen et al. 2012).

In the analyses of the total study sample, the association between repeated exposure to high ICT demands at work and suboptimal SRH remained stable even after adjustments for several potential cofounders. For instance, no obvious difference in the results was found when the analysis was additionally adjusted for job strain. This is interesting since previous results have implied an partial overlap between ICT demands at work and job strain (Stadin et al. 2016). However, the result in the present study may indicate that the ICT demands scale measures stressors in the work environment that have an independent impact on health-related outcomes, separate from the impact of job strain.

An interaction effect between ICT demands at work and sex was found, with men having a higher risk of developing suboptimal SRH after repeated exposure to high ICT demands at work. The reason for this result cannot be fully determined, but a hypothesis is related to the sex segregated labour market (Swedish Work Environment Authority 2016). Another possible partial explanation is related to a higher proportion of men that are working at distance to some extent, which mostly requires access to ICT (Swedish Work Environment Authority 2016).

A higher frequency of participants with high- and intermediate SEP reported consistently high ICT demands at work, than participants with low SEP. A higher frequency of high ICT demands at work in the high- and intermediate SES strata was also observed in previous cross-sectional analyses (Stadin et al. 2016). This result can partly be related to the characteristics of the used ICT demands at work scale, which might be more applicable to ICT-related stress in occupations with higher SEP, since this scale is rather office-oriented. However, only minor differences in the association between repeated exposure to high ICT demands at work and suboptimal SRH was observed between participants with high SEP and low SEP, and the interaction term between ICT demands at work and SEP was not significant. The reason of this result cannot be determined in the present study, but the result could potentially be related to a limitation in the ICT demands at work scale, that do not contrast ICT demands at work towards ICT-related resources. Possibly, there are social gradients in the amount of ICT-related resources (e.g., access to IT-support and influence over use of IT-systems (Day et al. 2012)), which in that case would have influenced the association between high ICT demands at work and suboptimal SRH with regard to SEP. Further development of the measurement of ICT-related stress at work to also include the dimension of ICT-related resources, is warranted and would make the measurement more in line with the JD-R model (Bakker and Demerouti 2007).

### Strengths and limitations

The present study has several strengths. The study contributes new information about prospective associations between ICT-related stress and a health-related outcome, which previously have been little explored in the scientific literature. By measuring repeated exposure to ICT demands at work, we were able to find that there was a difference between one single exposure to high ICT demands at work and repeated exposure to high ICT demands at work, with regard to suboptimal SRH. In the present study, a subjective health-related outcome was measured, which is an important complement to clinical health-related outcomes. This is because SRH is useful to measure the total subjective health status, which is not always captured when a single clinical diagnosis is measured. In addition, even though SRH is a subjective health-related measure, suboptimal SRH is predictive for serious clinical health-related outcomes and mortality (Singh-Manoux et al. 2007a, b).

The results were based on data from a large study population that was assumed to be representative for the working population in Sweden, which strengthens the generalisability of the results. The results provide an overview of the association between high ICT demands at work and suboptimal SRH in the general working population, but also separately in men and women, and in different SEP strata, to determine if the risk of suboptimal SRH is more elevated in any of these subgroups. Concerning the internal validity, the outcome variable, SRH, is a reliable and validated measure, which increases the internal validity (Singh-Manoux et al. 2007a, b). Likewise, the measures of job strain and social support (DCQ and DCSQ), are frequently used, reliable and validated measures (Karasek and Theorell 1992; Sanne et al. 2005). In addition, efforts have been made to control for several potential confounders, which reduces the influence of potential systematic bias, and strengthens the internal validity.

The present study has also some limitations. The ICT demands at work scale has no specified time duration, which makes it impossible to determine if the experience of stress was very temporary or had lasted for a long period. Consequently, in the analyses of repeated exposure to high ICT demands at work, no conclusion about exposure for a long period of time can be drawn. In addition, the ICT demands at work scale may not capture all potential aspects relevant to ICT-related stress. For instance, aspects such as lack of control, continuing learning expectations and ineffective communication, have been identified as important aspects of ICT-related stress elsewhere (Day et al. 2012).

Although SRH is a valid and frequently used health-related outcome variable, it has sometimes been criticised for being too unspecific, and a broader health index, e.g. SF-36 (Sullivan et al. 1995), could maybe have given more detailed information on the participants’ health status. Another consideration is that even though responders with suboptimal SRH at baseline were excluded, the risk of reversed causality cannot be fully excluded, due to the possibility that some of the participants might have developed suboptimal SRH between T1 and T2.

Considering the time aspect, the use of ICT has increased dramatically since the introduction of smartphones, tablets, etc. (Swedish Work Environment Authority 2015a), which chronologically happened after the first data collections in 2006 and 2008. When the results were calculated separately in study samples A (baseline 2006) and B (baseline 2008), the risk of suboptimal SRH after repeated exposure to high ICT demands at work was overall somewhat more pronounced in study sample B. This might imply that the prospective association between high ICT demands at work and suboptimal SRH would be even more elevated if conducted today. The potential influence of instrumentation bias due to over- or underestimation of the self-ratings of the exposure and outcome should also be considered, as well as the risk of residual confounding. In addition, the risk for over-adjustment in the regression models with adjustment for several covariates should be considered. It should also be acknowledged that additional aspects than those that were covered in the present study, could have impacted on the result if measured (e.g., personality traits and occupational category) (Grawitch et al. 2017). There is also a risk that the results are influenced by the healthy worker effect (Baillargeon 2001), since only people that were gainfully employed at the time of all three measurement were analysed.

## Conclusion

The present study indicated that exposure to high ICT demands at work measured at two points in time, 2 years apart, was associated with increased risk of developing suboptimal SRH at 4-years follow up after the baseline measurement. This association was modified by sex, and the risk was only present in men. However, the association between ICT demands at work and health-related outcomes should be further explored, preferably by measuring the exposure duration of ICT demands at work, along with associations with additional health-related outcomes (e.g., clinical outcomes).
